# Autoimmune Dysfunction Due to Severe Malaria

**DOI:** 10.7759/cureus.25458

**Published:** 2022-05-29

**Authors:** Aretha Kou, Jonathan Kirschen, Koravangala Sundaresh, Parth Desai

**Affiliations:** 1 Department of Internal Medicine, HCA Florida Trinity Hospital, Trinity, USA; 2 Department of Infectious Diseases, HCA Florida Trinity Hospital, Trinity, USA; 3 Department of Pulmonary and Critical Care Medicine, HCA Florida Trinity Hospital, Trinity, USA

**Keywords:** travel-related infection, infectious disease, plasmodium falciparum, severe plasmodium falciparum, pulmonary critical care, severe malaria, blackwater fever, cerebral malaria

## Abstract

Despite advances in treatment and prevention, malaria still carries significant morbidity and mortality. Cases of malaria in the United States are rare and cases of severe malaria, mostly attributable to *Plasmodium falciparum*, are even more uncommon. With the coronavirus disease 2019 (COVID-19) pandemic, there have been distractions in evaluation and diagnosis leading to a rise in cases and deaths. We present a case of autoimmune dysregulation and blackwater fever secondary to severe malaria, requiring multiple courses of antimalarial therapy. Careful travel history and prompt recognition and treatment facilitates improved patient survival and recovery.

## Introduction

In 2020, there were 241 million new malaria infections worldwide. Of those infections, 627,000 cases caused mortality [1]. From 2010 to 2015, approximately 2,000 cases of malaria were diagnosed in the United States annually [2]. Of those cases, 300 were severe and there were between 5-11 deaths per year [2]. Symptoms can be non-specific and diagnosis is by either rapid diagnostic test or microscopy. Severe malaria is primarily caused by *Plasmodium falciparum* and cases are defined by one or more of the following symptoms: jaundice, >5% parasitemia, neurological symptoms, renal failure, acute respiratory distress syndrome, or severe anemia [3]. Six *Plasmodium* species cause malaria infection in humans, with *P. falciparum *and *Plasmodium vivax *being the most common [4]. Despite optimal treatment, the mortality rate is still 10-20%, and almost 100% fatal if untreated [4]. Blackwater fever has recently been described in Ugandan children but was originally a rare and antiquated term that largely disappeared in the early 20th century [5]. It is classically characterized by massive intravascular hemolysis secondary to *P. falciparum* infection and quinine use with a possible genetic link to glucose-6-phosphate dehydrogenase deficiency [5]. However, there have been reports of blackwater fever associated with artesunate use [6]. The etiology is still not fully understood [5]. To date, there have been few reports about blackwater fever with autoimmune dysregulation caused by malaria. In this paper, we present a case of a 62-year-old male diagnosed with blackwater fever secondary to severe malaria subsequently leading to autoimmune dysregulation.

## Case presentation

**Figure 1:**
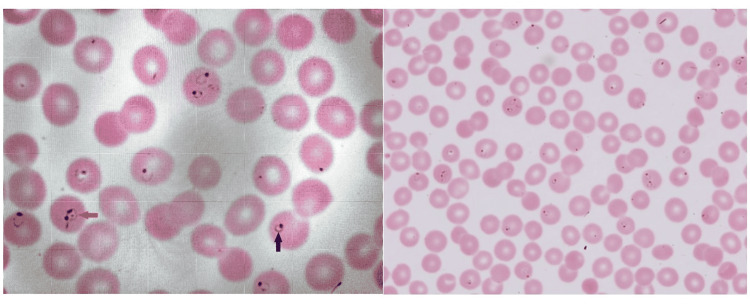
(A) Peripheral blood smear showing classic rings (black arrow) and head-phoned rings (red arrow); (B) Peripheral blood smear showing Falciparum parasitemia >30%

A 62-year-old male with a past medical history of hypertension, hyperlipidemia, and diabetes presented to the emergency department complaining of dehydration, headache, and chills. He took two coronavirus disease 2019 (COVID-19) tests at home, the most recent test being three days prior to admission. The first test indicated positive and the second showed negative. He received both doses of the Pfizer vaccine in March 2021. Home medications included metformin, amlodipine, losartan, simvastatin, aspirin, and ibuprofen. Initial vital signs were notable for a temperature of 37.8°C and a heart rate of 105 beats per minute. Respiratory rate, blood pressure, and oxygen saturation in room air were within normal limits. The physical exam was unremarkable. Laboratory tests were significant for lactic acid of 3.6 mmol/L, creatinine of 2.1 mg/dL, total bilirubin of 5.1 mg/dL, and platelet count of 37,000/uL. Urinalysis was positive for protein, ketones, nitrites, bilirubin, urobilinogen, mucus, and bacteria. He was admitted for management of sepsis and presumed COVID-19 infected. Infectious Disease was consulted for severe sepsis. He initially reported recent travel to London, United Kingdom. Later, his wife reported his travel to Nigeria to visit family during the same 12-day trip. He returned to the United States 11 days prior to admission. The next day he became confused and computed tomography of the head showed no acute process. There was a concern for cerebral malaria with his new impaired mental status. Peripheral smear obtained in light of travel history showed greater than 30% parasitemia consistent with malaria along with classic rings and head-phoned rings (Figures 1A, 1B). The diagnosis of *P. falciparum* malaria was confirmed by polymerase chain reaction (PCR) of whole blood and by whole blood peripheral smear using QuantStudio 12K Flex Real-Time PCR system (Thermo Fisher Scientific, Waltham, Massachusetts, United States). COVID-19 PCR test was negative.

The CDC was contacted and he was started on artesunate. On day three, he was transferred to the intensive care unit for further management of decompensating mental status, renal function, and liver function. During his prolonged hospital stay, he developed acute renal failure secondary to acute tubular necrosis requiring dialysis and acute hemolytic anemia requiring blood transfusion. The patient had relapsing fevers up to 39.4˚C with waxing and waning mentation. After traumatic nasogastric tube insertion, he developed diffuse nasal bleeding unrelieved by nasal packing. He was intubated for concern of risk of hemorrhagic aspiration. Bleeding was successfully controlled with bilateral nasal tampons. Esophagogastroduodenoscopy performed for melena showed no acute signs of bleeding. A percutaneous endoscopic gastrostomy (PEG) tube was placed on day 24 for tube feeding. There was concern regarding hemophagocytic lymphohistiocytosis (HLH) given his cytopenia, fever, hypertriglyceridemia (>354mg/dL) off of propofol, ferritin greater than 6000 ng/dL, hepatomegaly, and significantly increased IL-2 level of 4031 units/mL. Using the HScore for reactive hemophagocytic syndrome, he had a >99% probability of HLH with a score of 247. However, improving thrombocytopenia and lack of persistent fever, hypofibrinogenemia, or splenomegaly went against a diagnosis of HLH. Total bilirubin, aspartate aminotransferase (AST), and alanine aminotransferase (ALT) increased to 34 mg/dL, 649 unit/L, and 259 unit/L, respectively, suggesting liver failure. The serology panel showed positive Epstein Barr virus IgM, IgG, and nuclear antigen antibody. Anti-smooth muscle antibody was positive and liver biopsy was recommended, but the patient’s family declined. Pertinent serology and immunology tests are summarized in Table 1.

**Table 1:** Serology results, immunology results, and parasitemia load during hospital course LKM-1: liver kidney microsome type 1; ANCA: antineutrophil cytoplasmic antibodies; P-ANCA: perinuclear antineutrophil cytoplasmic antibodies; C-ANCA: antineutrophil cytoplasmic autoantibody, cytoplasmic; COVID-19: coronavirus disease 2019; NAA: nucleic acid amplification; SARS-CoV-2: severe acute respiratory syndrome coronavirus 2; PCR: polymerase chain reaction; EBV: Epstein-Barr virus; Ag: antigen

Serology and Immunology Markers					Results				
Soluble Interleukin-2					4,031 (223-710 units/mL)				
Anti-LKM-1 Antibody					3.4 (0-20)				
Rheumatoid Factor					< 8.6 (0.0-12.0)				
Antinuclear Antibodies					Negative				
C-ANCA					< 0.2 (0.0-0.9)				
P-ANCA					< 0.2 (0.0-0.9)				
Anti-Mitochondrial Antibody					0.67 (<0.90)				
Anti-Smooth Antibody					86 on Day 20, 141 on Day 28 (0-19)				
Thyroid Peroxidase					< 9.0 (<9.0)				
Glomerular Base Membrane Antibody					6 (0-20)				
COVID-19 (NAA), SARS-CoV-2 (PCR)					Positive NAA (Day 2), Negative PCR (Day 6)				
EBV Capsid Ag IgG Antibody					3.8 (0.0-0.8)				
EBV Capsid Ag IgM Antibody					1.8 (0.0-0.8)				
EBV Early Antigen Antibody					0.30 (0.0-0.8)				
EBV Nuclear Antigen Antibody					7.90 (0.0-0.8)				
Hepatitis A IgM Antibody					Negative				
Hepatitis Bs Antigen					Negative				
Hepatitis Bs Antibody					Positive A				
Hepatitis B Core IgM Antibody					Negative				
Hepatitis C Antibody					Negative				
HCV DNA					Negative				
HIV-1 p 24					Negative				
HIV (1&2) Ag/Ab					Negative				
HIV 1&2 Antibody Rapid					Negative				
IgG					1485				
IgA					209				
IgM					138				
Immunoglobulins					Immunofixation pattern unremarkable				
Parasitemia (Reference: Negative)									
Day 2	Day 4	Day 5	Day 6	Day 7	Day 8	Day 9	Day 25	Day 27	Day 28
30%	<5%	1.9%	0.9%	1.7%	0.6%	0.0%	0.0%	0.1%	0.0%

Parasite percentage eventually decreased to 0%; however, he required two courses of artesunate, artemether/lumefantrine, and artemisinin for persistent parasitemia (Table 1). The patient’s renal function, mentation, liver enzymes, and anemia gradually improved. He was extubated and downgraded to the progressive care unit. He began tolerating an oral diet and the PEG tube was no longer indicated. He was discharged home with home healthcare for hemodialysis. A follow-up clinic visit four weeks after discharge showed normal mentation and slowly improving renal function on hemodialysis.

## Discussion

Severe malaria is rare in the United States and blackwater fever is even more uncommon. In the current case, the patient had jaundice, >5% parasitemia, cerebral malaria, renal failure, and severe anemia consistent with severe malaria. Diagnostic challenges in this patient included delay in diagnosis due to the relative rarity of malaria in the United States as well as initial presumed COVID-19 diagnosis. During the COVID-19 pandemic, there have been distractions in evaluation and diagnosis leading to a rise in cases and deaths. In 2019, there were 227 million global malaria cases. In contrast, in 2020 there were 241 million new malaria cases worldwide [1]. Lessons from this case include close examination of travel history and close follow-up on information, as patients may not be forthcoming about recent travel history. Other diagnostic challenges included contacting the CDC for appropriate medications as well as lack of availability of in-house smears in a community hospital to monitor parasitemia. Indeed, similar cases of delay in diagnosis as well as obtaining artesunate have been reported [7].

Unique points of this case include autoimmune dysfunction likely secondary to cytokine upregulation and inflammation. Although artemisinin derivatives have been documented to cause mild (1-4%) elevation of serum aminotransferase, autoantibodies have not been described [8]. Thus, positive anti-smooth muscle antibodies may be secondary to autoimmune hepatitis. However, liver biopsy is required for definitive diagnosis. It has also been recognized that acute infectious processes, both bacterial and viral, can cause smooth muscle antibody positivity [9]. Positivity for EBV IgM, IgG, and EBV nuclear antigen antibody suggested reactivation as the patient did not show symptoms of infectious mononucleosis [10]. Research has shown that *P. falciparum* antigens can induce lytic activation of previously latent EBV B cell infection during acute malarial infection [11]. Similarly, his constellation of symptoms was thought less likely to be HLH given his improvement. The increase in IL-2 was most likely multifactorial due to causes including renal failure, liver failure, and immunosuppression. Although cases of malaria-associated immunosuppression have been reported, research into the etiology is fragmented with no clear mechanism explained [12]. Malaria affects multiple mechanisms of the host immune system, including suppressing T cell proliferation and antibody production by affecting humoral and cellular responses [12]. The patient met criteria for the syndrome of malarial hepatopathy, defined as a bilirubin >2.5 times upper limit of normal (ULN) with transaminase elevation >3 ULN [8,13]. Although previous studies have noted mild transaminase elevation due to artemisinin treatment, one study did not find an association with timing of treatment regimen and peak transaminase elevation [13]. Further study is needed to differentiate between malaria-induced liver injury versus treatment induced transaminase elevation.

Despite the advances of medicine, malaria still carries significant morbidity and mortality if not promptly recognized and treated. In particular, there is a mortality rate of 15-20% in cerebral malaria even when treated [7]. Precautions can be taken including prophylactic antimalarials such as doxycycline, chloroquine, and mefloquine that are recommended by the CDC [3]. In 2021, the first malaria vaccine, and the first vaccine against any parasitic disease, was approved by the WHO for children [1]. Although this is a great advancement in the eradication of malaria, further progress must be made in prevention and treatment.

## Conclusions

Malaria is still a serious and potentially fatal disease worldwide despite the approval of the first malaria vaccine for children in 2021 and the availability of prophylactic anti-malarials. Severe and uncommon complications such as autoimmune dysregulation and blackwater fever may result. With the focus on the COVID-19 pandemic for the past few years, education on the prevention and treatment of malaria has been lagging. It is important to promptly recognize risk factors and treat malaria symptoms.

## References

[1] (2021). World malaria report 2021. World Malaria Report 2021.

[2] Rosenthal PJ, Tan KR (2019). Expanded availability of intravenous artesunate for the treatment of severe malaria in the United States. Am J Trop Med Hyg.

[3] Hwang J, Cullen KA, Kachur SP, Arguin PM, Baird JK (2014). Severe morbidity and mortality risk from malaria in the United States, 1985-2011. Open Forum Infect Dis.

[4] Plewes K, Leopold SJ, Kingston HW, Dondorp AM (2019). Malaria: what's new in the management of malaria?. Infect Dis Clin North Am.

[5] Shanks GD (2017). The multifactorial epidemiology of blackwater fever. Am J Trop Med Hyg.

[6] Jauréguiberry S, Ndour PA, Roussel C (2014). Postartesunate delayed hemolysis is a predictable event related to the lifesaving effect of artemisinins. Blood.

[7] Rodriguez JA, Roa AA, Leonso-Bravo AA, Khatiwada P, Eckardt P, Lemos-Ramirez J (2020). A case of Plasmodium falciparum malaria treated with artesunate in a 55-year-old woman on return to Florida from a visit to Ghana. Am J Case Rep.

[8] Artemisinin. Livertox: Clinical And Research Information On Drug-induced Liver Injury [Internet].

[9] Kanakoudi-Tsakalidis F, Cassimos C, Papastavrou-Mavroudi T (1979). Mechanisms of smooth muscle antibody production: a clinical study in children with infections, haemolytic syndromes, and idiopathic thrombocytopenic purpura. J Clin Pathol.

[10] De Paschale M, Clerici P (2012). Serological diagnosis of Epstein-Barr virus infection: problems and solutions. World J Virol.

[11] Chêne A, Donati D, Guerreiro-Cacais AO (2007). A molecular link between malaria and Epstein-Barr virus reactivation. PLoS Pathog.

[12] Calle CL, Mordmüller B, Singh A (2021). Immunosuppression in malaria: do Plasmodium falciparum parasites hijack the host?. Pathogens.

[13] Woodford J, Shanks GD, Griffin P, Chalon S, McCarthy JS (2018). The dynamics of liver function test abnormalities after malaria infection: a retrospective observational study. Am J Trop Med Hyg.

